# The Effect of Digital Literacy Training on Physical Activity App Acceptance and Behavioral Intentions Among Older Women: An Experimental Study

**DOI:** 10.3390/ijerph23040489

**Published:** 2026-04-13

**Authors:** Silvija Baubonytė

**Affiliations:** Department of Sport and Tourism Management, Lithuanian Sports University, LT-44221 Kaunas, Lithuania; silvija.baubonyte@lsu.lt

**Keywords:** digital literacy, physical activity apps, behavioral intentions, older women, UTAUT2, active aging

## Abstract

**Highlights:**

**Public health relevance—How does this work relate to a public health issue?**

- Physical activity apps support activity monitoring and health behavior change; however, low digital literacy remains a significant barrier to their effective use among older adults.
- This study evaluates whether app-specific digital literacy training improves app acceptance and strengthens technology-based health promotion and healthy aging.

**Public health significance—Why is this work of significance to public health?**

- An app-specific digital literacy intervention significantly improved technology acceptance and self-reported behavioral intentions to use physical activity apps as well as intentions to engage in physical activity.
- The findings demonstrate that digital literacy education can empower older women to use health technologies and strengthen health-supportive behavioral intentions in the short term.

**Public health implications—What are the key implications or messages for practitioners, policy makers and/or researchers in public health?**

- App-specific digital literacy education should be incorporated into community-based health promotion programs targeting older women.
- Addressing digital competence is essential for reducing age-related digital inequalities and supporting sustainable healthy aging strategies.

**Abstract:**

Physical activity apps offer significant potential to promote physical activity and active aging; however, their acceptance among older adults remains limited, often due to insufficient digital literacy. This study aimed to examine whether targeted, app-specific digital literacy training can improve eHealth literacy, acceptance of physical activity apps, and behavioral intentions among older women, drawing on the Unified Theory of Acceptance and Use of Technology 2 (UTAUT2) extended with a personal innovativeness construct. A total of 63 older women (M = 67.0, SD = 4.6) were randomly assigned to an experimental (*n* = 32) or control group (*n* = 31). The experimental group participated in a nine-week digital literacy training focused on practical use of physical activity apps. Measures were collected before and after the intervention. Data were analyzed using repeated-measures MANOVA and ANOVAs. A significant Group × Time interaction was observed for technology acceptance (Wilks’ Λ = 0.41, *F* (7, 54) = 11.14, *p* < 0.001, *η*_p_^2^ = 0.59). The experimental group showed significant improvements across all measured constructs. The largest effects were found for eHealth literacy (*η*_p_^2^ = 0.39) and intention to use physical activity apps (*η*_p_^2^ = 0.24). App-specific digital literacy training can enhance technology acceptance and support physical activity–related intentions among older women, highlighting its potential to reduce digital barriers and promote active aging. The findings reflect short-term, self-reported changes in technology acceptance and behavioral intentions.

## 1. Introduction

Physical activity is one of the key factors supporting health, independence, and quality of life among older adults and is widely recognized as a core component of active aging [1,2]. It is considered one of the most important means of preventing chronic diseases, as well as a significant factor in maintaining or improving functional and cognitive abilities [1,3,4], thereby contributing to healthy aging [5].

Despite the well-established benefits of physical activity, many older adults face barriers that limit their ability to remain physically active. These barriers are multifactorial and include health-related, motivational, environmental, and socioeconomic factors, such as health problems, lack of time, low motivation, limited access to facilities, and financial constraints [6,7,8,9]. For these reasons, increasing attention has been given to digital approaches that may help overcome such barriers, including physical activity apps, as they offer accessible and resource-efficient ways to monitor and manage physical activity. Research has also demonstrated that these apps can serve as accessible and effective tools for promoting physical activity and supporting self-regulation [9,10], as well as improving functional capacity and physical fitness in later life [11], thereby contributing to a better quality of life among older adults [12].

Although the use of physical activity apps among older adults has increased in recent years [1], their acceptance and continued use remain uneven and depend on psychological and technological factors. One of the most widely applied theoretical approaches to the analysis of technology acceptance is the Unified Theory of Acceptance and Use of Technology 2 (UTAUT2) [13], which posits that behavioral intention to use technology is shaped by performance expectancy, effort expectancy, social influence, facilitating conditions, hedonic motivation, habit, and price value. Subsequent research has extended this model by incorporating personal innovativeness [14]. Although innovativeness is often considered more characteristic of younger users, recent studies indicate that older adults may also demonstrate curiosity and a willingness to experiment with sport and health apps [15,16]. Research focusing on older populations consistently identifies the core UTAUT2 factors as important determinants of technology acceptance in later life [17,18,19,20,21]. While these models demonstrate strong predictive value, evaluations of technology acceptance may be influenced by broader factors, including age, health, psychological factors, technology design, social and infrastructural conditions, as well as prior experience and digital literacy [22,23,24,25]. Among these, digital literacy appears particularly important, as its limitations are closely intertwined with a range of individual, social, and contextual barriers that jointly influence technology use among older adults [26].

According to the Digital Economy and Society Index (DESI), levels of digital literacy decline significantly from the age of 55 onwards [27]. Older adults often have limited prior experience with digital technologies and face both psychological barriers and insufficient digital literacy [22,23,25]. Limited digital literacy is associated with lower self-confidence, higher levels of technology-related anxiety, and more negative attitudes toward technology [28,29]. Evidence suggests that low digital literacy is often associated not only with health-related issues, lack of support, and perceived barriers, but also with resource constraints, limited knowledge, and technological factors, such as ease of use and perceived usefulness [26].

Moreover, empirical evidence suggests that the acceptance of health technologies may differ by gender [30]. While some studies report that men experience higher levels of technology-related anxiety than women [31], other findings indicate that women more often exhibit lower acceptance of health technologies because they perceive these tools as more complex and effort-demanding [32]. In addition, women’s intentions to use technology appear to depend more strongly on self-efficacy, trust in technology, and existing digital skills [33]. Furthermore, broader research on the digital divide indicates that women, particularly in older age groups, may experience lower levels of digital literacy and reduced access to digital resources compared to men, which may further contribute to disparities in the use of digital health technologies [34,35,36]. Taken together, these findings suggest that older women may represent a relevant target group for targeted digital literacy interventions.

Digital literacy can be considered a key factor shaping technology acceptance and use, including health technologies and physical activity apps [19,21,37,38]. In the health context, it is often discussed in relation to eHealth literacy, which is defined as the ability to locate, understand, and apply digital health information [39]. Research indicates that eHealth literacy is associated with technology use both directly and indirectly through technology acceptance factors [19,21,38], and low levels of these competencies may limit access to health information and negatively affect health outcomes [31].

In response to the challenges of the digital divide, increasing attention has been devoted to strengthening digital competencies among older adults [40]. Although digital literacy training programs can reduce technology-related anxiety [41,42] and increase trust in digital tools [43,44], their evaluations most often emphasize general digital skills rather than competencies directly related to the use of health technologies or physical activity apps [45,46]. Moreover, older adults may encounter both internal and external barriers during training that constrain effective skill development [47], highlighting the need for targeted interventions aligned with specific technological and behavioral goals [46,48].

Finally, systematic reviews indicate that evaluations of eHealth literacy interventions have primarily focused on informational, motivational, and behavioral outcomes [49], while technology acceptance mechanisms are still examined inconsistently and often rely on earlier frameworks such as the Technology Acceptance Model (TAM) [41], which does not capture the full range of determinants currently reflected in UTAUT2. Although a limited number of experimental studies have investigated the effects of digital skills training on technology acceptance using UTAUT-based frameworks [49], these interventions have typically focused on general smartphone use and have not been situated within the context of health- or physical activity–related apps. Moreover, prior research has rarely examined whether such training-induced changes in technology acceptance translate into intentions related to health-promoting behaviors. Consequently, a significant research gap remains regarding whether structured, app-specific digital literacy training can influence UTAUT2 technology acceptance factors in the context of physical activity apps, as well as intentions to engage in physical activity, particularly among older women. As physical activity apps have been identified as potentially effective tools for promoting physical activity, digital literacy training may serve as an important strategy for increasing their acceptance among older women.

Therefore, the aim of this study is to examine whether digital literacy training can act as an intervention influencing UTAUT2 technology acceptance factors, intentions to use physical activity apps, and intentions to engage in physical activity among older women. Specifically, we tested whether the intervention produced differential pre–post changes (Group × Time) in eHealth literacy, UTAUT2 acceptance constructs, intention to use physical activity apps, and intention to be physically active. The study provides empirical evidence on the effects of a digital literacy training intervention on technology acceptance factors in the context of physical activity app use among older women with prior app experience, thereby contributing to broader active aging objectives. Accordingly, the findings reflect changes in post-experience technology acceptance.

## 2. Materials and Methods

### 2.1. Participants

The required sample size was determined based on statistical power analysis parameters (α = 0.05; 1 − β = 0.80), to detect between-group differences, following established methodological guidelines [50]. Based on these parameters, a minimum of 31 participants per group was required.

Three inclusion criteria were applied: (1) age 55 years or older; (2) prior experience using physical activity apps; and (3) engagement in at least minimal PA. The age threshold of 55 years was defined based on the European Digital Economy and Society Index (DESI), which indicates that a marked decline in digital literacy levels begins from this age onward [27]. Women were selected as the target population based on evidence indicating that they tend to exhibit lower acceptance of health technologies, often perceiving them as more complex and effort-demanding [32], and that their intentions to use technology are more strongly influenced by existing digital skills [33]. In addition, research on the digital divide indicates that women, particularly in older age groups, may have lower levels of digital literacy and reduced access to digital resources, which may further limit their engagement with digital technologies [34,36]. While findings on gender differences are not entirely consistent, older women may represent a particularly relevant target group for digital literacy interventions.

The requirement of prior experience with physical activity apps was applied to ensure that participants were able to provide informed evaluations of technology acceptance constructs, as meaningful assessment of UTAUT2-related factors presupposes at least minimal firsthand interaction with the technology. Accordingly, eligibility was restricted to “ever-users” (current or former users), although participants were not required to be active users at the time of the study.

Engagement in at least minimal physical activity (PA) was required to distinguish purposeful app use for activity monitoring and goal pursuit from purely exploratory or curiosity-driven use, which may be irregular and unrelated to sustained PA engagement. Minimal PA was operationalized as participation in intentional, planned physical activity at least once per week. This criterion was considered an indicator that participants used physical activity apps in a context relevant to the study aims.

Participants were recruited using purposive and snowball sampling techniques [51]. Initial contact was established with the coordinator of a University of the Third Age, who facilitated access to potential participants and enabled the presentation of the study. Subsequently, participants were invited to share information about the study with relatives or acquaintances who met the inclusion criteria.

In total, 67 older women were randomized. Complete pre–post data were available for 63 participants (experimental *n* = 32; control *n* = 31), who were included in the final analyses. The mean age at baseline was 67.0 years (SD = 4.6). All participants reported being physically active and having prior experience using physical activity apps. Detailed demographic characteristics, physical patterns, and app use characteristics, including duration and frequency of use, are presented in Table 1.

### 2.2. Study Design and Procedures

The study was conducted between 21 January and 28 March 2025 and followed a randomized controlled trial design with a three-stage pre–post structure: (1) baseline assessment, (2) educational intervention, and (3) post-intervention assessment. Baseline data were collected on 21 January, when all participants were invited to the university, informed about the study procedures, provided written informed consent, and completed the initial quantitative survey. The study was registered at *ClinicalTrials.gov* (Identifier: NCT07515898) on 7 April 2026.

Of the 69 individuals assessed for eligibility, two were excluded after screening (one did not meet the age criterion and one was male). The remaining 67 participants were randomized. Following the baseline assessment, participants were randomly assigned to either the experimental group (*n* = 34) or the control group (*n* = 33) using a stratified block randomization procedure [52]. Randomization sequences were generated using a computer-based tool prior to allocation. Blinding was not feasible due to the educational nature of the intervention. Randomization was performed with a 1:1 allocation ratio using blocks of four and was stratified according to two criteria: (1) age group (57–66 years vs. 67–77 years) and (2) prior experience with physical activity apps (current users vs. former users with prior experience). These stratification variables were selected because both age and prior app usage experience may meaningfully influence evaluations of technology acceptance.

Four participants (two per group) did not complete the post-intervention assessment; therefore, complete pre–post data were available for 63 participants (experimental *n* = 32; control *n* = 31), who were included in the final analyses.

**Figure 1:**
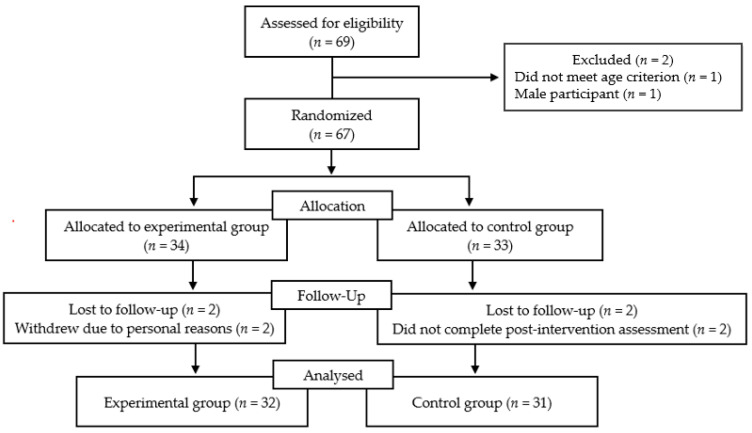
CONSORT flow diagram of participant recruitment, allocation, and analysis.

Participants in the experimental group received the digital literacy educational intervention, whereas the control group continued their usual daily activities without receiving any educational program during the study period. Both groups completed identical assessments at baseline and post-intervention. Participant flow throughout the study is presented in Figure 1.

The final analyzed sample remained balanced (experimental group: *n* = 32; control group: *n* = 31). No significant differences between groups were observed at baseline across stratification variables (age category: *p* = 0.580; prior app usage experience: *p* = 0.879) or across any study variables (all *p* > 0.05).

### 2.3. Instruments

The instruments used in this study were based on previously established and widely used measures and were adapted for the Lithuanian context.

eHealth literacy was assessed using the eHealth Literacy Scale (eHEALS) developed by Norman and Skinner (2006), which measures individuals’ perceived knowledge, confidence, and skills in locating, evaluating, and applying electronic health information for health-related purposes [53]. The scale consists of eight items rated on a five-point Likert scale ranging from 1 (strongly disagree) to 5 (strongly agree). Total scores are calculated by summing item responses, yielding a possible score range from 8 to 40, with higher scores indicating greater perceived eHealth literacy [54].

Acceptance of physical activity apps was assessed using a questionnaire based on the Unified Theory of Acceptance and Use of Technology 2 (UTAUT2) proposed by Venkatesh et al. (2012) [13], supplemented with the personal innovativeness construct as proposed by Farooq et al. (2017) [14]. This extended framework enables the assessment of technology acceptance and behavioral intentions through the following constructs: performance expectancy, effort expectancy, social influence, facilitating conditions, hedonic motivation, habit, and personal innovativeness. The price value construct was excluded, as the physical activity apps considered in this study were available free of charge.

Each construct was measured using items adapted from prior UTAUT2-based research. The number of items per construct followed commonly used configurations in the literature: performance expectancy (4 items), effort expectancy (4 items), social influence (5 items), facilitating conditions (4 items), hedonic motivation (3 items), habit (3 items), personal innovativeness (3 items), and behavioral intention (4 items in total). Behavioral intention was operationalized as two outcomes: (1) intention to use physical activity apps and (2) intention to engage in physical activity. Intention to use physical activity apps was measured using three items adapted from UTAUT2, whereas intention to engage in physical activity was assessed with a single item reflecting planned engagement in physical activity. All items were rated on a five-point Likert scale (1 = strongly disagree to 5 = strongly agree). Composite scores were computed as the mean of the corresponding items; the single-item physical activity intention was analyzed separately.

All questionnaire items were translated into Lithuanian and culturally adapted using a standard forward–backward translation procedure [55].

All multi-item subscales demonstrated very good internal consistency. Cronbach’s alpha (α) coefficients ranged from 0.832 to 0.963, while McDonald’s omega (ω) coefficients ranged from 0.865 to 0.964. The behavioral intention to engage in physical activity was assessed using a single item and therefore reliability coefficients were not calculated for this outcome. The overall scale reliability across multi-item constructs was also high (α = 0.879; ω = 0.885), indicating strong internal coherence.

Because the questionnaire had not previously been validated in the Lithuanian context, it was first adapted and psychometrically validated in an independent sample prior to the implementation of the present experimental study. Exploratory (EFA) and confirmatory factor analyses (CFA) were conducted in an independent cross-sectional sample of Lithuanian older adults (*n* = 414). The validation sample (*n* = 414) was randomly divided into two equal subsamples (*n* = 207 each), with EFA conducted on the first subsample and CFA performed on the second. The data met the assumptions for factor analysis (KMO > 0.60; Bartlett’s test of sphericity, *p* < 0.001). The EFA yielded a total explained variance exceeding 50%, with factor loadings ranging from 0.622 to 0.991. The CFA supported good model fit (χ^2^ (1364) = 3215, *p* < 0.001; χ^2^/df = 2.36; RMSEA = 0.078; CFI = 0.953; TLI = 0.950; SRMR = 0.062).

### 2.4. Educational Intervention

The educational intervention for the experimental group was conducted between 28 January and 25 March 2025. The duration and structure of the program were determined based on a review of relevant scientific literature and the scope of the planned training content. Previous studies indicate that digital literacy interventions vary considerably in length, ranging from several weeks to up to one year [56,57]. Importantly, the effectiveness of such programs depends on aligning intervention duration with the number and complexity of targeted competencies, allowing sufficient time for gradual skill acquisition.

In the present study, the intervention consisted of nine thematic sessions addressing the key competencies required for effective use of physical activity apps. Sessions were delivered once per week, a frequency considered appropriate for supporting digital skill development and maintaining learning motivation among older adults [57]. The nine-week duration is also consistent with prior research identifying this timeframe as effective for digital literacy development [58].

Each session lasted 60 min and was conducted face-to-face at university facilities. Sessions followed a consistent weekly schedule, which contributed to consistently high attendance rates throughout the program (90–100%).

The educational program comprised nine structured sessions designed to develop digital literacy skills related explicitly to physical activity app use. The intervention emphasized practical, hands-on learning and progressive skill development. An overview of the program structure, including session topics, instructional methods, and learning objectives, is provided in Table 2.

The educational program was developed based on the core competencies required for the effective use of physical activity apps and was delivered by the researcher, who had prior experience in both educational practice and the use of apps. All sessions followed a pre-established instructional plan to ensure consistency in content and teaching methods. At the beginning of the program, participants’ needs were assessed to evaluate their existing skills and to adapt the content, pace, and instructional strategies accordingly [46].

Given that older adults benefit from clear, structured, and visually supported learning materials [59], participants received 24-page printed guides tailored to different smartphone manufacturers (Apple, Samsung, Huawei, and Xiaomi). Instructions were presented step by step and accompanied by illustrative screenshots. The guides were used during sessions and served as additional support for homework tasks. After completion of the study, the same materials were also provided to participants in the control group.

During the first session, participants were introduced to the goals of the program, the importance of PA, and different types of physical activity apps. Subsequent sessions followed a predefined thematic sequence. Each session adhered to a standardized structure: approximately 10 min were devoted to reviewing homework assignments, 15 min to explanation and demonstration of new content, and the remaining time to hands-on practice. As information retention may decline with age, regular practice and homework assignments are considered essential for effective learning [60]. Accordingly, participants received homework tasks after each session aimed at reinforcing newly acquired skills, identifying difficulties, and increasing independence and self-confidence.

To ensure that tasks were clearly understood [60,61], homework instructions were explained verbally during sessions and subsequently sent to participants via email, together with lecture slides and supplementary materials. Throughout the program, participants received individual assistance and consultations from the researcher as needed.

Instructional methods were selected in accordance with evidence-based recommendations for older adult learning, taking into account psychological, physical, and technological factors [47]. All instructional content and the learning environment were designed to be clear, visually supported, patient, and encouraging, thereby fostering a safe and supportive learning atmosphere [46,59,62]. As participants did not speak or understand English, the practical component of the intervention relied exclusively on physical activity apps with Lithuanian language support, including Health, Walk15, and Google Fit.

This educational structure was designed to support the development of practical skills for independent use of physical activity apps and to create a positive and supportive learning experience.

### 2.5. Ethics

The study was conducted in accordance with established ethical principles for social science research and complied with the Declaration of Helsinki. Prior to data collection, ethical approval was obtained from the University Social Research Ethics Committee (Approval No. SMTEK-271, approved on 29 June 2024).

All participants were fully informed about the purpose, procedures, and conditions of the study, as well as their right to withdraw at any time without any negative consequences. Written informed consent was obtained from all participants prior to their inclusion in the study.

Participant anonymity and confidentiality were strictly ensured. All data were used exclusively for scientific purposes, analyzed in aggregated form, and stored in a manner that prevented the identification of individual participants. To enable pre- and post-intervention comparisons while preserving anonymity, each participant was assigned a unique identification code that did not contain directly identifiable personal information.

Given that the study involved older adults, particular attention was paid to presenting information in a clear and accessible manner and to safeguarding participants’ psychological comfort throughout the entire research process.

### 2.6. Statistical Data Analysis

Statistical analyses were performed using IBM SPSS Statistics version 29.0.1.0 (IBM Corp., Armonk, NY, USA). Prior to the primary analyses, data distribution was examined by assessing skewness and kurtosis. According to Hair et al. (2022), data may be considered normally distributed when skewness and kurtosis values fall within the range of −1 to +1; however, values within ±2 are commonly accepted in social science research [63]. In the present study, the assumption of normality was therefore considered satisfied when skewness and kurtosis values did not exceed ±2.

Descriptive statistics were calculated for all variables, including means (M), standard deviations (SD), skewness (Sk), and kurtosis (Ku). Baseline group equivalence was assessed using independent-samples *t*-tests, with differences considered non-significant at *p* > 0.05.

To evaluate the effects of the digital literacy training program, a 2 × 2 repeated-measures multivariate analysis of variance (MANOVA) was conducted to examine the interaction between group (experimental vs. control) and time (pre-intervention vs. post-intervention). The assumptions of multivariate analysis, including homogeneity of covariance matrices, were examined and met.

When a significant multivariate interaction effect was observed, follow-up univariate repeated-measures ANOVAs were conducted for each priori defined acceptance construct. Given the limited number of theoretically specified outcomes, findings were interpreted alongside effect sizes (*η*_p_^2^). As a sensitivity analysis, *p*-values were additionally examined using the Holm–Bonferroni procedure; conclusions remained unchanged.

eHealth literacy and the two behavioral intention outcomes were analyzed using separate 2 × 2 repeated-measures ANOVAs.

Effect sizes were calculated using partial eta squared (*η*_p_^2^) and interpreted with reference to Cohen’s (1988) benchmarks for proportion-of-variance effect sizes, where values of approximately 0.01, 0.06, and 0.14 indicate small, medium, and large effects, respectively [64].

To further explore the relationships among study variables, Pearson correlation analyses were performed to examine associations between eHealth literacy, UTAUT2 acceptance constructs, and behavioral intentions. These analyses were considered exploratory in nature and are presented in the Supplementary Materials.

## 3. Results

An analysis of the descriptive statistics for the total sample prior to the intervention revealed moderate baseline levels across all study variables (Table 3).

Prior to the intervention, participants reported generally moderate levels across most study variables, with comparatively higher scores for performance expectancy, hedonic motivation, social influence, intention to use apps and be physically active. Lower scores were reported for effort expectancy, habits, facilitating conditions, and innovativeness. Overall eHealth literacy reflected a moderate baseline level.

Following the intervention, mean scores in the total sample tended to increase across measured constructs. The most pronounced improvements were evident in eHealth literacy, as well as in effort expectancy, facilitating conditions, social influence, habit, and performance expectancy. After the intervention, behavioral outcomes also showed positive changes in both behavioral intention to use physical activity apps and behavioral intention to be physically active.

Skewness and kurtosis values for all variables were within the acceptable range (−2 to +2), indicating that the assumption of normality was met (Table 3).

No significant group differences were found for performance expectancy (*t* (59) = −0.416, *p* = 0.679, *d* = −0.11), effort expectancy (*t* (59) = 0.595, *p* = 0.554, *d* = −0.12), facilitating conditions (*t* (59) = 1.261, *p* = 0.212, *d* = 0.32), hedonic motivation (*t* (59) = 0.627, *p* = 0.533, *d* = 0.16), habit (*t* (59) = 0.478, *p* = 0.634, *d* = 0.12), social influence (*t* (59) = 0.961, *p* = 0.340, *d* = 0.25) and innovativeness (*t* (59) = −0.387, *p* = 0.700, *d* = −0.10). Likewise, no significant baseline differences were found between the groups for behavioral intention to use physical activity apps (*t* (59) = −0.492, *p* = 0.625, *d* = −0.13), or behavioral intention to engage in physical activity (*t* (59) = −0.412, *p* = 0.682, *d* = −0.11). Finally, the experimental and control groups did not differ significantly in overall eHealth literacy scores (*t* (59) = 1.407, *p* = 0.165, *d* = 0.36). These findings confirm that the experimental and control groups were comparable at baseline, supporting the internal validity of the intervention study.

A statistically significant Group × Time interaction was observed for the UTAUT-related acceptance constructs (Wilks’ Λ = 0.41, *F*( 7, 54) = 11.14, *p* < 0.001, *η*_p_^2^ = 0.59), indicating that changes over time differed significantly between the experimental and control groups.

Intervention effects for each acceptance construct, as well as for eHealth literacy and behavioral intention outcomes, are presented in Table 4.

A strong and statistically significant Group × Time interaction effect was observed for eHealth literacy (*F* (1, 61) = 38.13, *p* < 0.001, *η*_p_^2^ = 0.39). The experimental group demonstrated a substantial increase in eHealth literacy scores from pre-test to post-test, whereas the control group showed virtually no change over time.

Significant Group × Time interaction effects were also found for all UTAUT-related constructs—performance expectancy (*F* (1, 61) = 8.01, *p* = 0.006, *η*_p_^2^ = 0.12), effort expectancy (*F* (1, 61) = 11.92, *p* < 0.001, *η*_p_^2^ = 0.17), facilitating conditions (*F* (1, 61) = 14.68, *p* < 0.001, *η*_p_^2^ = 0.20), hedonic motivation (*F* (1, 61) = 15.63, *p* < 0.001, *η*_p_^2^ = 0.21), habit (*F* (1, 61) = 8.09, *p* = 0.006, *η*_p_^2^ = 0.12), social influence (*F* (1, 61) = 4.07, *p* = 0.048, *η*_p_^2^ = 0.06), innovativeness (*F* (1, 61) = 7.29, *p* = 0.009, *η*_p_^2^ = 0.11), with increases primarily observed in the experimental group.

Significant intervention effects were additionally observed for behavioral intention to use physical activity apps (*F* (1, 61) = 18.98, *p* < 0.001, *η*_p_^2^ = 0.24), and behavioral intention to be physically active (*F* (1, 61) = 7.16, *p* = 0.010, *η*_p_^2^ = 0.11), indicating increased intentions among participants exposed to the intervention.

Overall, these findings indicate that the digital literacy intervention produced consistent and statistically significant improvements across technology acceptance, eHealth literacy, and behavioral intention outcomes in the experimental group, while the control group remained largely stable over time. Effect sizes ranged from medium to large, with the strongest effects observed for eHealth literacy (*η*_p_^2^ = 0.39) and behavioral intention to use physical activity apps (*η*_p_^2^ = 0.24).

To further explore post-intervention associations among study variables within the experimental group (*n* = 32), correlation results are presented in Supplementary Table S1. eHealth literacy was significantly positively associated with performance expectancy (*r* = 0.544, *p* < 0.01), effort expectancy (*r* = 0.642, *p* < 0.01), hedonic motivation (*r* = 0.404, *p* < 0.05), and innovativeness (*r* = 0.418, *p* < 0.05), but was not significantly related to facilitating conditions, social influence, habit, or behavioral intentions. Behavioral intention to use physical activity apps was significantly correlated with performance expectancy (*r* = 0.377, *p* < 0.01), effort expectancy (*r* = 0.622, *p* < 0.01), facilitating conditions (*r* = 0.762, *p* < 0.01), and social influence (*r* = 0.731, *p* < 0.01). The full correlation matrix is presented in the Supplementary Materials.

## 4. Discussion

This study examined the effects of a structured, app-specific digital literacy intervention on the acceptance of physical activity apps among older women. The findings demonstrated a consistent positive impact of the intervention across all UTAUT2-related acceptance constructs, as well as on eHealth literacy and behavioral intentions to use apps and to engage in physical activity. These results indicate that technology acceptance is not shaped solely through prolonged exposure or habitual use, but can also be meaningfully enhanced through educational programs that integrate knowledge acquisition with confidence-building and hands-on practice. Strengthening digital literacy may contribute to improved acceptance of physical activity apps and more favorable behavioral intentions among older women.

In this study, the educational intervention was grounded in previous research emphasizing that effective digital literacy education requires consideration of older learners’ needs, capabilities, and challenges related to technology use, as well as the application of age- and experience-appropriate teaching methods [46,59,62]. Accordingly, the training program was structured to align with participants’ age, prior experience, technological abilities, and self-identified needs. The results further demonstrated that following the educational intervention, eHealth literacy among participants in the experimental group increased significantly, whereas no comparable changes were observed in the control group. These findings are consistent with previous research indicating that educational interventions can be effective in improving eHealth literacy [43,44,65,66]. However, it is important to consider that the study sample consisted of individuals with prior experience using physical activity apps and relatively high levels of education. Although their digital literacy was moderate, both prior experience and education may have been associated with greater engagement with the intervention and the observed positive outcomes.

Although the scientific literature suggests that digital and eHealth literacy training programs often last from several weeks to one year or longer [56,57], the present findings indicate that a nine-week educational program may be sufficient to achieve meaningful improvements in eHealth literacy; however, the long-term sustainability of these effects remains to be established.

Among the app acceptance–related outcomes, one of the most pronounced improvements was observed in performance expectancy. Following the intervention, participants demonstrated a clearer perception of the benefits provided by physical activity apps, indicating an enhanced recognition of their usefulness. This pattern aligns with previous research showing that hands-on engagement and functional understanding of technology play a critical role in shaping perceived usefulness [17,18,19].

Effort expectancy also increased, indicating that the apps were perceived as easier to use after the training. This finding supports previous evidence that digital literacy enhances user competence and perceived usability of mHealth technologies [67]. As perceived complexity is a major factor contributing to technology avoidance [68], reduced perceived effort indicates that the intervention effectively lowered both psychological and functional barriers to use. Hands-on learning likely played a central role in helping participants understand how apps operate and thereby reduce perceived difficulty, consistent with previous research highlighting the role of structured learning, active practice, and confidence building in digital literacy development among older adults [46,69].

Improvements were also observed in facilitating conditions, despite no objective changes in access to equipment or internet connectivity during the study period. Previous research indicates that facilitating conditions are closely related to perceived competence [70]. However, correlation analysis based on post-intervention data in the experimental group revealed no significant association between eHealth literacy and facilitating conditions (Supplementary Table S1). In contrast, facilitating conditions were significantly correlated with performance expectancy, effort expectancy, hedonic motivation, and social influence—constructs that also improved following the intervention. This may reflect subjective shifts in participants’ perceptions, related to more favorable evaluations of usefulness, ease of use, and emotional experience. When app use is perceived as easy and beneficial, older users may also be more likely to evaluate their available resources more positively [20], a pattern that is consistent with the findings of the present study. However, in this study, the findings from the correlational analyses should be interpreted as associations that may reflect underlying relationships rather than causal effects.

The results also revealed a significant increase in hedonic motivation, indicating an improvement in user experience. These findings complement previous research showing that higher levels of digital literacy are associated with more positive emotional evaluations of physical activity apps [18]. Increased literacy and perceived competence may reduce previously experienced usage barriers, thereby diminishing negative emotional reactions toward technology. Importantly, correlation analysis further indicated that hedonic motivation was significantly associated with both performance expectancy and effort expectancy (Supplementary Table S1). These relationships indicate that hedonic motivation may be associated not only with ease of use but also with perceived usefulness and the effort required to use the technology.

Among all examined outcomes, changes in habit formation were particularly relevant from a practical perspective. Although habit development is known to require sustained practice and experience [13], the structured training sessions and homework assignments provided participants with repeated opportunities to use physical activity apps in everyday contexts. This finding aligns with previous studies showing that habits strengthen when technologies are repeatedly used in real-life situations [71]. The observed variability further suggests that habit formation is a highly individualized process influenced by prior experience and personal motivation.

The significant increase in innovativeness offers additional insight into the psychological impact of the intervention. While innovativeness is often considered a relatively stable personality trait [72], the observed changes suggest that enhanced digital competence may be associated with greater openness to new technologies. This interpretation is consistent with post-intervention correlation analysis within the experimental group, which showed that eHealth literacy was significantly associated with innovativeness (Supplementary Table S1). This perspective is in line with previous research indicating that digital literacy development may reduce technology-related anxiety [41,42], strengthen trust in technology [43,44], and increase motivation to use digital tools [42,66,73]. Although technology-related anxiety was not directly assessed in this study, increased personal innovativeness may reflect greater psychological comfort and confidence in interacting with digital tools, rather than solely a stable dispositional trait.

Social influence exhibited the smallest change among the examined constructs, although the effect remained statistically significant. Previous studies suggest that higher levels of digital literacy may be associated with an enhanced ability to interpret, evaluate, and selectively apply social recommendations rather than with uncritical reliance on them [18,21]. As digital competence increases, individuals may become better equipped to assess the relevance and credibility of social cues, balancing autonomy with openness to external input. In the present study, however, social influence in the experimental group was not directly associated with eHealth literacy but was significantly correlated with effort expectancy and facilitating conditions (Supplementary Table S1). This pattern may suggest that evaluations of social influence are more closely associated with perceived ease of use and the adequacy of available resources than with individual digital skills. Importantly, the social influence construct encompasses not only the opinions of significant others but also active endorsement, acceptance of recommendations, and responsiveness to others’ behaviors [74]. When close social contacts support and recommend a technology, users are more likely to evaluate it favorably and recognize its usefulness [75].

Importantly, the intervention resulted in a significant increase in behavioral intentions to use physical activity apps, whereas no such changes were observed in the control group. Previous research indicates that digital literacy is associated with intentions to use technology both directly and indirectly through technology acceptance constructs [18,19,37,43]. In the present study, no significant direct association between eHealth literacy and behavioral intentions to use physical activity apps within the experimental group was observed. However, the intervention significantly improved technology acceptance constructs, which were also significantly associated with behavioral intentions. Taken together, the correlation pattern suggests that both eHealth literacy and technology acceptance constructs may be associated with behavioral intentions, particularly effort expectancy and facilitating conditions, with these relationships interpreted as exploratory. These findings reflect changes in technology acceptance following prior app use and do not capture initial adoption processes. Accordingly, the present study focuses on post-experience technology acceptance rather than first-time engagement among complete non-users, which represents a conceptually distinct process.

Finally, the intervention also led to a medium-sized increase in intentions to engage in physical activity, suggesting its practical relevance. Although the program primarily focused on the use of physical activity apps, improved digital competencies and more favorable evaluations of app acceptance may be associated with stronger intentions to engage in physical activity and enhanced perceived behavioral control. This finding is consistent with recent research indicating that digital literacy may indirectly relate to physical activity and improve health-related outcomes [76]. Together, these findings suggest that digital literacy, technology acceptance, and behavioral intentions represent related but conceptually distinct constructs. In the present study, improvements were observed across all three domains, while exploratory correlations indicated possible associations among them. However, in this study, intention to engage in physical activity was assessed using a single-item measure, which may limit the reliability and conceptual depth of this outcome and therefore warrants cautious interpretation.

In addition to these findings, this study has several strengths. First, it examined a structured, app-specific digital literacy intervention rather than general digital skills training, thereby providing more targeted insights into the use of physical activity applications. Second, the study applied the UTAUT2 framework in an experimental design, allowing for the assessment of changes in technology acceptance constructs over time. Third, by focusing on older women, a group often facing greater barriers to technology use, the study contributes to research on an underrepresented population in digital health. Fourth, the prior adaptation and validation of the measurement instrument in a Lithuanian sample strengthen the validity and cultural appropriateness of the assessed constructs. Finally, the findings extend current knowledge by linking digital literacy development with technology acceptance constructs and behavioral intentions related to physical activity.

### Limitations and Future Research

First, the study included only older women. Although previous research suggests that women more frequently encounter challenges related to technology use and are therefore considered an important target group for digital interventions, it remains unclear whether the obtained results can be generalized to men or other age groups. Furthermore, although baseline eHealth literacy levels were moderate, the majority of participants held higher education degrees, which may have been associated with greater responsiveness to the intervention and a faster learning process. Therefore, caution is warranted when generalizing the findings to populations with lower levels of educational attainment.

Second, the study relied on self-report questionnaires to assess technology acceptance and behavioral intentions. Although such instruments are widely used in technology acceptance research, they do not permit objective verification of actual app use or physical activity behavior. While app usage was monitored during the program, it was not treated as a primary outcome because the intervention design (hands-on sessions and homework) required frequent app interaction, making post-intervention increases difficult to interpret as independent behavioral change beyond training compliance. Accordingly, the findings should be interpreted as changes in acceptance constructs and intentions rather than verified changes in physical activity behavior. In addition, intention to engage in physical activity was assessed using a single-item measure, which may limit the reliability and conceptual depth of this outcome and therefore warrants cautious interpretation.

Third, participants in the control group did not receive any comparable educational input during the intervention period; therefore, it is difficult to fully distinguish the specific effects of the app-focused digital literacy training from potential non-specific factors such as attention, participant–researcher contact, or expectancy effects (i.e., the absence of an attention-matched control condition). In addition, the effects of the educational intervention were assessed one week after the completion of the training. Although this allows for the evaluation of short-term intervention outcomes, this study design does not permit conclusions regarding the long-term sustainability of the observed changes.

Finally, the requirement of prior app experience was conceptually aligned with the UTAUT2 framework, which assumes evaluative judgments based on at least minimal system interaction. However, the findings may not directly apply to complete non-users, who may represent a particularly relevant target group for such interventions.

Beyond the specific context of physical activity app use, the findings of this study contribute to broader discussions on digital inclusion, active aging, and preventive eHealth strategies. As health promotion increasingly relies on digital solutions, ensuring that older adults possess the skills and confidence to engage with such technologies represents an important public health challenge. Interventions that combine digital literacy education with practically oriented, behavior-supporting applications may play a significant role in reducing age-related digital inequalities and supporting healthy aging trajectories.

Future research should include “never-users” of physical activity apps and examine adoption readiness or initial acceptance (rather than post-experience acceptance), in order to determine whether app-specific digital literacy training also facilitates first-time uptake. In addition, longitudinal designs are needed to examine whether improvements in technology acceptance translate into sustained long-term app use and objectively measured changes in physical activity behavior. Studies involving mixed-gender samples and different age cohorts would further strengthen generalizability. Finally, as correlation analyses revealed complex relationships among digital literacy, UTAUT2 constructs, and behavioral intentions, larger samples and structural equation modeling (SEM) should be employed to test potential direct and indirect (mediational) mechanisms more rigorously.

## 5. Conclusions

This study demonstrates that a structured digital literacy training program tailored to physical activity app use can meaningfully enhance eHealth literacy, improve acceptance of physical activity apps, and strengthen behavioral intentions to use digital tools among older women. Importantly, the intervention was associated not only with improved technology acceptance but also with increased behavioral intentions to engage in physical activity, suggesting its potential to support physical activity–related behavioral intentions among older women.

The observed improvement in eHealth literacy suggests that strengthening individuals’ ability to access, understand, and apply digital health information may contribute to more favorable technology acceptance and stronger behavioral intentions. In the present study, these outcomes improved concurrently following the intervention. While additional analyses indicated associations among these constructs, these findings should be interpreted cautiously, as no mediational pathways were formally tested. Overall, the findings support the value of structured, age-appropriate educational interventions for reducing technology-related barriers and promoting digital inclusion and active aging.

Future research should examine the long-term sustainability of these effects and investigate whether similar intervention approaches are effective across more diverse populations, including individuals of different ages and genders, as well as those with no prior experience in using physical activity apps.

## Figures and Tables

**Table 1:** Demographic characteristics, physical activity patterns, and physical activity app use characteristics of participants in the experimental and control groups (*n* = 63).

		Experimental (*n* = 32)	Control (*n* = 31)	Total (*n* = 63)
Age	57–60	4 (12.5)	3 (9.7)	7 (11.1)
	61–64	5 (15.6)	5 (16.1)	10 (15.9)
	65–68	12 (37.5)	11 (35.5)	23 (36.5)
	69–73	7 (21.9)	9 (29.0)	16 (25.4)
	74–77	4 (12.5)	3 (9.7)	7 (11.1)
Education	Non-university higher education	7 (21.9)	4 (12.9)	11 (17.5)
	Bachelor’s degree	15 (46.9)	13 (41.9)	28 (44.4)
	Master’s degree	10 (31.3)	13 (41.9)	23 (36.5)
	Doctoral degree	0 (0.0)	1 (3.2)	1 (1.6)
Marital Status	Cohabiting with a partner	1 (3.1)	2 (6.5)	3 (4.8)
	Married	17 (53.1)	17 (54.8)	34 (54.0)
	Divorced	6 (18.8)	1 (3.2)	7 (11.1)
	Widowed	5 (15.6)	8 (25.8)	13 (20.6)
	Single	3 (9.4)	3 (9.7)	6 (9.5)
PA Frequency (days/week)	1–2 days/week	9 (28.1)	5 (16.1)	14 (22.2)
	3–4 days/week	15 (46.9)	13 (41.9)	28 (44.4)
	5–6 days/week	6 (18.8)	3 (9.7)	9 (14.3)
	Daily	2 (6.3)	10 (32.3)	12 (19.0)
PA Duration (minutes/session)	Less than 30 min	3 (9.4)	3 (9.7)	6 (9.5)
	30–60 min	19 (59.4)	18 (58.1)	37 (58.7)
	60–90 min	8 (25.0)	9 (29.0)	17 (27.0)
	More than 90 min	2 (6.3)	1 (3.2)	3 (4.8)
PA App Use Status	Current user	17 (53.1)	15 (48.4)	32 (50.8)
	Former user	15 (46.9)	16 (51.6)	31 (49.2)
App Use Duration	Not currently using	15 (46.9)	16 (51.6)	31 (49.2)
	3–6 months	5 (15.6)	3 (9.7)	8 (12.7)
	6–11 months	4 (12.5)	6 (19.4)	11 (17.5)
	1–2 years	6 (18.8)	4 (12.9)	10 (15.9)
	3–5 years	2 (6.3)	1 (3.2)	3 (4.8)
	More than 5 years	0 (0.0)	1 (3.2)	1 (1.6)
App Use Frequency (times/week)	Not currently using	15 (46.9)	16 (51.6)	31 (49.2)
	1–2 times/week	5 (15.6)	4 (12.9)	8 (12.7)
	3–4 times/week	7 (21.9)	4 (12.9)	11 (17.5)
	5–6 times/week	4 (12.5)	6 (19.4)	11 (17.5)
	Daily	1 (3.1)	1 (3.2)	2 (3.2)

Note. Values are expressed as *n* (%); percentages for experimental and control groups are calculated within groups, and percentages for the total column are based on the overall sample.

**Table 2:** Structure, Teaching Methods, and Learning Goals of the Digital Literacy Training Program.

No.	Topics	Methods	Goals
**1.**	Types, Opportunities, and Benefits of PA apps	explanation; discussion	To understand the importance of physical activity, recognize ways to engage in it, identify types and functions of PA apps, choose suitable apps according to one’s needs, and evaluate their usefulness.
**2.**	Use of PA Apps: Data Privacy and Protection	explanation; discussion; demonstration	To understand the importance of data protection, evaluate the safety of using PA apps, and make informed decisions regarding one’s personal data.
**3.**	Use of PA Apps: Managing Privacy Settings	explanation; demonstration; practice; feedback; homework	To understand the role of privacy settings and be able to check, adjust, and personalize them according to one’s needs.
**4.**	Searching, Selecting, and Downloading PA Apps	explanation; demonstration; practice; collaboration; feedback; homework	To know how to search for, select, and download suitable PA apps, as well as install, open, and delete them.
**5.**	Functions of PA Apps: Creating a User Profile and Setting Goals	explanation; demonstration; practice; feedback; homework	To be able to create a user account, log in, switch accounts, set activity goals based on one’s abilities, find goal-setting features, and adjust them.
**6.**	Functions of PA Apps: Recording and Tracking PA	explanation; demonstration; practice; feedback; homework	To locate the activity tracking function, select activities, understand activity indicators, and start, pause, and finish tracking sessions.
**7.**	Functions of PA Apps: Searching and Analyzing Activities	explanation; demonstration; practice; collaboration; feedback; homework	To find previously recorded activities; access weekly, monthly, and yearly summaries; interpret indicators in relation to one’s health.
**8.**	Functions of PA Apps: Social Interaction and Community Features	explanation; demonstration; practice; collaboration; homework	To understand the purpose and benefits of social features; navigate the community section; join or create groups; add or remove members.
**9.**	Functions of PA Apps: Summary, Discussion and Feedback	discussion; practice	To integrate all previously learned functions during a practical consolidation session and reflect on learning through group discussion.

**Table 3:** Means (M), standard deviations (SD), skewness (Sk), and kurtosis (Ku) of the study variables before and after the intervention (*n* = 63).

Variables	Before Intervention			After Intervention		
	M ± SD	Sk	Ku	M ± SD	Sk	Ku
eHealth literacy	22.30 ± 6.10	−0.10	0.75	26.90 ± 6.65	−0.64	1.13
Performance expectancy	3.98 ± 0.54	−0.01	0.30	4.25 ± 0.55	0.08	−0.43
Effort expectancy	2.80 ± 0.81	0.24	1.25	3.60 ± 0.65	−0.25	0.48
Facilitating conditions	3.12 ± 0.65	−0.59	−0.21	3.98 ± 0.67	−0.62	1.10
Hedonic motivation	3.53 ± 0.85	0.07	−0.52	3.57 ± 0.87	−0.13	−0.54
Habit	2.98 ± 0.93	0.39	−0.25	3.57 ± 0.75	0.34	0.91
Social influence	3.50 ± 0.87	−0.16	0.27	3.89 ± 0.88	−0.30	−0.65
Innovativeness	2.74 ± 1.07	0.26	−0.40	2.76 ± 0.84	0.09	−0.83
Behavioral intention to use PA apps	3.60 ± 0.70	0.24	0.03	3.94 ± 0.66	0.05	−0.60
Behavioral intention to be PA	3.80 ± 0.70	−0.07	−0.16	3.95 ± 0.78	−0.34	−0.30

Note. M = mean; SD = standard deviation; Sk = skewness; Ku = kurtosis.

**Table 4:** Pre- and post-intervention means and standard deviations (M ± SD) and Group × Time interaction effects for study variables.

Variables	Experimental Group		Control Group		Group × Time Interaction		
	Pre-Test (M ± SD)	Post-Test (M ± SD)	Pre-Test (M ± SD)	Post-Test (M ± SD)	*F*	*p*	*η*_p_^2^
eHealth literacy	21.94 ± 5.94	29.84 ± 4.23	22.32 ± 6.63	22.36 ± 7.14	38.13	<0.001 ***	0.39
Performance expectancy	4.01 ± 0.49	4.40 ± 0.52	3.95 ± 0.59	3.90 ± 0.62	8.01	0.006 **	0.12
Effort expectancy	2.73 ± 0.73	3.73 ± 0.53	2.86 ± 0.90	3.10 ± 0.74	11.92	<0.001 ***	0.17
Facilitating conditions	3.02 ± 0.60	3.88 ± 0.67	3.23 ± 0.69	3.40 ± 0.69	14.68	<0.001 ***	0.20
Hedonic motivation	3.46 ± 0.70	3.89 ± 0.68	3.60 ± 1.00	3.42 ± 0.91	15.63	<0.001 ***	0.21
Habit	2.93 ± 0.83	4.03 ± 0.90	3.04 ± 1.03	3.1 ± 1.14	8.09	0.006 **	0.12
Social influence	3.40 ± 0.93	3.71 ± 0.82	3.61 ± 0.79	3.65 ± 0.78	4.07	0.048 *	0.06
Innovativeness	2.67 ± 1.00	2.95 ± 0.81	2.67 ± 0.91	2.57 ± 1.01	7.29	0.009 **	0.11
Behavioral intention to use PA apps	3.69 ± 0.66	4.09 ± 0.60	3.60 ± 0.82	3.62 ± 0.77	18.98	<0.001 ***	0.24
Behavioral intention to be PA	3.77 ± 0.73	4.19 ± 0.65	3.84 ± 0.64	3.70 ± 0.84	7.16	0.010	0.11

Note. M ± SD—mean and standard deviation; *η*_p_^2^ = partial eta squared; * *p* ≤ 0.05; ** *p* ≤ 0.01; *** *p* ≤ 0.001.

## Data Availability

The data presented in this study are available from the corresponding author upon reasonable request.

## Supplementary Materials

The following supporting information can be downloaded at: https://www.mdpi.com/article/10.3390/ijerph23040489/s1, Table S1: Post-intervention correlations among study variables (experimental group, N = 32).

## Abbreviations

The following abbreviations are used in this manuscript:

PA	Physical activity
UTAUT	Unified theory of acceptance and use of technology
