# Supplementary material for: The Effect of Digital Literacy Training on Physical Activity App Acceptance and Behavioral Intentions Among Older Women: An Experimental Study
Source: Int J Environ Res Public Health. 2026 Apr 13;23(4):489. doi: 10.3390/ijerph23040489 (PMC13116906; doi:10.3390/ijerph23040489)
Supplement: Supplementary file 1 [file ijerph-23-00489-s001.zip › ijerph-4185866-supplementary.pdf]

Supplementary Table S1. Post-intervention correlations among study variables (experimental group, *n* = 32)

	**1**	**2**	**3**	**4**	**5**	**6**	**7**	**8**	**9**	**10**
1. eHealth literacy	-	-	-	-	-	-	-	-	-	-
2. Performance expectancy	**.544****	-	-	-	-	-	-	-	-	-
3. Effort expectancy	**.642****	**.480****	-	-	-	-	-	-	-	-
4. Facilitating conditions	.325	**.541****	**.638****	-	-	-	-	-	-	-
5. Hedonic motivation	**.404***	.326	**.532****	**.403***	-	-	-	-	-	-
6. Habit	.023	.108	.089	-.034	.218	-	-	-	-	-
7. Social influence	.168	.181	**.477****	**.702****	.302	-.021	-	-	-	-
8. Innovativeness	**.418***	.238	.299	.190	**.533****	.139	-.003	-	-	-
9. Behavioral intentions to use apps	.261	**.377***	**.622****	**.762****	.194	.071	**.731****	-.024	-	-
10. Behavioral intentions to be physically active	.185	**.366***	.346	**.656****	.033	.164	**.525****	.117	**.732****	-

Note. *$p \leq .05$; **$p \leq .01$
